# Synthesis, characterization, and application of N-CNT/1-(2-Hydroxyethyl)-3-methylimidazolium dicyanamide as a green nanocatalyst for the sulfur removal from light oils

**DOI:** 10.1016/j.heliyon.2024.e24073

**Published:** 2024-01-04

**Authors:** Seyed Mohammad Reza Shoja, Majid Abdouss, Raheleh Saeedirad

**Affiliations:** Department of Chemistry, Amirkabir University of Technology, P.O. Box 15875-4413, Tehran, Iran

**Keywords:** N-doped carbon nanotubes, Imidazolium based ionic liquid, Desulfurization, Adsorption kinetics

## Abstract

Adsorptive desulfurization of light fuels is sustainable due to its ambient operation and reusability of exhausted adsorbents. In this study, 1-(2-hydroxyethyl)-3-methylimidazolium dicyanamide [HEMIM][DCA] IL was synthesized and utilized to modify N-doped carbon nanotubes (CNTs) to produce N-CNT/[HEMIM][DCA] as a green hybrid adsorbent. The adsorbent was characterized using XRD, FE-SEM, FTIR, BET, and TGA. It was indicated that the N-CNT treatment with [HEMIM][DCA] IL resulted in decreased crystallinity with the cubic and rod-shaped morphology and harsh surfaces and curved edges. The absence of shifts or variations in FTIR peaks of starting materials and N-CNT/[HEMIM][DCA] suggested that neither component was affected by chemical interactions. The adsorption capacity of N-CNT and N-CNT/[HEMIM][DCA] was 54.3 mg/g and for 83.6 mg/g for 50 ppm BT, respectively. Saturated with BT, the adsorbent's performance was decreased at high BT concentrations. The adsorption isotherms provided an understanding of interactions of BT with sorbent surface which follows the Langmuir model for N-CNT/[HEMIM][DCA] and N-CNT. The kinetics of BT adsorption on N-CNT/[HEMIM][DCA] was fitted with second-order kinetic model with the decreased adsorption ratio over time due to pore saturation. 25 % reduction of the adsorption capacity was obtained after two recycling cycles of the adsorbent (62.5 mg/g). N-CNT/[HEMIM][DCA] showed good recyclability and potential as a promising BT adsorbent.

## Graphical abstract

**Figure:**
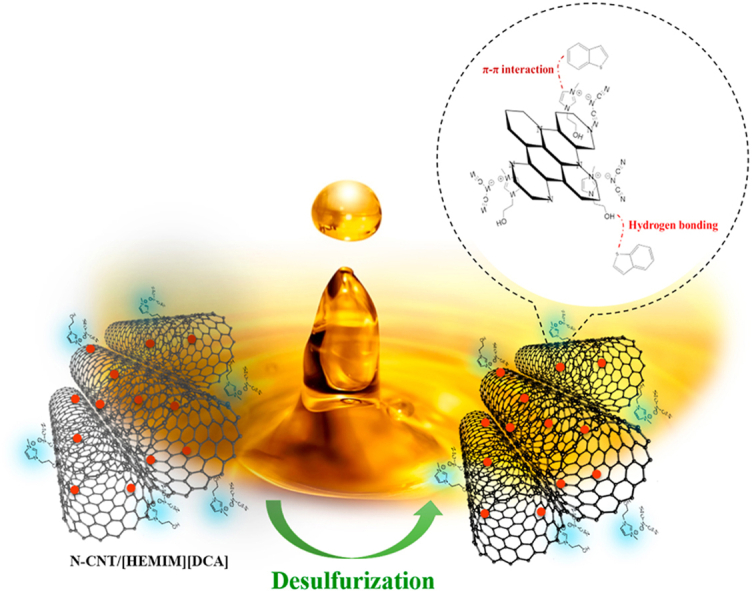

## 1 Introduction

The sulfur content of light oils has been gradually reduced across the globe by environmental policies to reduce atmospheric pollution [[1], [2], [3]]. Producing sulfur-free fuel can produce cleaner environments and healthier societies [4,5]. Sulfuric compounds in liquid fuels are highly reactive, volatile, and toxic, causing severe environmental concerns of toxin emissions, catalyst poisoning, ecological destruction, and corrosion, which can damage chemical instruments and pipelines [6,7].

To remove sulfur compounds and reduce sulfur content to less than about 1 ppm under mild conditions, adsorptive desulfurization (ADS) has gained attention because of its ease of handling, low price, substantial capacity, selectivity, and market prospects [[8], [9], [10]]. Many adsorbents can be used in the gaseous and liquid fuels' desulfurization process, including C-based adsorbents , zeolites, ionic liquids, and metal oxides [[11], [12], [13], [14], [15], [16], [17]].

Among them, several unique properties of C-based adsorbents (activated and porous C [18,19], graphene [[20], [21], [22]], and C nanotube (CNT) [[23], [24], [25]]), including high surface areas, chemical and thermal endurances make them good candidates for the removal of pollutants [26,27]. The ability of activated C to adsorb has been improved using several methods. CNTs incorporate nitrogen atoms in the form of pyrrolic and pyridinic nitrogen-containing bonds in the basal plane [28]. Compared to pristine CNTs, N-doped CNTs (N-CNTs) nitrogen sources release electrons [29]. Using interactions of either polar or acidic nature, doping is valuable for adsorption. S interaction with CNT is enhanced by pyridinic nitrogen, and capacitance is enhanced by pyrrolic nitrogen [30,31].

Recently, dispersed C nanoparticles were used to adsorb thiophenic compounds selectively [28]. According to Daraee et al. the use of N-CNT/ZIF-8 adsorbent improved the adsorptive capacity of the desulfurization process (81.2 mg/g) paralleled to the pristine ZIF-8 (69.1 mg/g) [32]. Also, a model diesel fuel was treated with adsorption using a carbon-based nanotube (M-CNT) and nanofiber (M-CNF) for BT removal with maximum adsorption of 43 % and 35 %, respectively [33]. Furthermore, CNT/TiO_2_ was synthesized and evaluated with the initial removal of BT (45 %) and thiophene (55 %), resulting in excellent interactions with organosulfur compounds [34]. Oxidative desulfurization of petrol products was investigated using decorated cobalt oxide-modified carbon nanotubes, and only 0.025 mg/l of the synthesized material could remove 68 % of sulfur impurities [35].

The “designer solvents” known as Ionic Liquids (ILs) have engrossed significant consideration due to their unique features [[36], [37], [38]] to enhance and modify the solid supports to produce nanocomposites. Nanocomposites are good candidates for adoptive process [[39], [40], [41]]. As a result of their chemical stability, non-volatility, and ease of workshop in the liquid state, ILs are excellent alternatives to traditional extraction media [42,43]. It is becoming increasingly popular to use ILs for sulfur compound extraction due to their operating in mild conditions, low consumption of energy, and environmental affability [44,45].

Immobilizing ILs on solid supports [[46], [47], [48]] can avoid their limitations of high cost, viscosity, and difficulty in recycling [49,50], such as polyether-based ILs for extractive gasoline desulfurization, with 84.7 %, 91.4 %, and 81.0 % desulfurization rates for BT, DBT, and 4,6-dimethyl dibenzothiophene (4,6-DMDBT) respectively [51]. Imidazolium-based ionic liquids were also utilized to eliminate sulfur from coal combined with H_2_O_2_ (30 %) based on chemically oxidative desulfurization (16.76 %) [52]. Dexin Sun et al. investigated solvent-sulfur interaction and indicated that the hybrid solvents, including ILs, display improved extraction desulfurization performance [53]. Extractive desulfurization (EDS) of internal concentrate fuels using ILs and profound eutectic solvents was investigated. The results revealed the high efficiency of ILs, especially imidazolium and pyridinium-based ILs in EDS [54,55]. Also, β-cyclodextrin-reduced graphene oxide-multiwall carbon nanotubes (IL@β-CD-rGO-MWCNTs), was utilized for the swift elimination of bentazon at pH 6.0 with a maximum adsorption capacity of 125.00 mg/g [56]. Mao et al. (2023) have recently discovered amphiphilic polyoxometalate-based poly(ionic liquid) copolymers known as 3,3′-methylenebis(1-vinylimidazol) bromine with a hydrophobic compound called [3–alkyl–1–vinylimidazolium] bromine for the desulfurization efficiency of up to 100 % across 0–50 °C for dibenzothiophene (DBT) [57].

Based on the reported methods, no specific reports are discussed the potential of ILs for improving the performance of N-doped CNTs as sulfur adsorbents. In this regard, this research is aimed to synthesize an imidazolium-based IL 1‐(2‐hydroxyethyl)‐3‐methylimidazolium dicyanamide [HEMIM][DCA] [42]. We prepared the novel green hybrid, N-CNT/[HEMIM][DCA], using the solvothermal method in the first step. Then, the hybrid product was used to desulfurize the Benzothiophene through adsorptive desorption. In the third step, thermodynamic and kinetic models were investigated for desulfurization.

## 2 Experimental

### 2.1 Materials

Commercially existing starting materials were utilized without additional purification. Methylene chloride (≥99 %), acetone (≥99 %), sodium dicyanamide (≥96 %), ammonia (≥99 %), 2-methylimidazole, benzothiphene (≥99 %), 1-Propanol (≥99 %) and silica-alumina catalyst support were purchased from 10.13039/100004334Merck Chemical Company. Sigma-Aldrich provided the CNTs. We also purchased ethane (99.99 %) and argon (99.999 %) from Merck. Pre-distilled deionized water was utilized in all chemical reactions.

### 2.2 Preparation process of nitrogen-doped carbon nanotube (N-CNT)

As previously described [58,59], using an ethane/argon/ammonia mixture on an alumina-supported iron catalyst, the chemical vapor deposition (CVD) technique can be utilized to attain an identical distribution of nitrogen atoms. The iron loading was set at 25 wt%. Ammonia was added to the reactant mixture as a nitrogen source. The carbon source and carrier gases were ethane and argon, respectively. The temperature ranges between 650 °C and 850 °C during the synthesis process. Then, residual alumina support is dispersed by treating the N-CNTs in a 20 wt% soda solution at 110 °C for 24 h. A neutral pH was achieved after several washes with deionized water. Aqua mixture was used to remove the residual iron catalyst after water washing for 17 h at 110 °C.

### 2.3 Preparation process of [HEMIM][DCA]IL

Typically, the solution was prepared for 1 g of [HEMIM][Cl] [42] 0.56 g sodium dicyanamide in 20 ml of acetone. Under the atmosphere of N_2_ gas and at room temperature, the mixture was stirred for two days in a condenser. Methylene chloride was used to dilute and filter the residual condensed liquid. A vacuum chamber at 70 °C (under 10-1 Pa) was utilized for 48 h after the solvent evaporation of the [HEMIM][DCA] IL product.

### 2.4 Preparation process of N-CNT/[HEMIM][DCA]

CNTs were modified as reported previously [60,61]. Under vacuum conditions (70 °C), N-CNT (1 g) was suspended in [HEMIM][DCA] (0.71 g) in 10 ml of ethanol. After sonicating for 3 h, stirring overnight, and centrifuging at 10000 rpm, the mixture was filtered. After washing with deionized water, the precipitate was dehydrated at 100 °C.

### 2.5 Desulfurization conditions

The desulfurization process was performed by employing a model fuel (1-propanol and n-octane) possessing 500 ppm BT. The ratio of S-contents of BT was measured by a gas chromatography detector (GC-8355 SCD, Agilent, USA) in the model fuel.

The features of the 30 m column can be described as a fused silica-wall coated open tube (WCOT) column with a diameter of 0.32 mm, covered with methyl silicone (with a thickness of 4 μm), contained the 0.1–2.0-μL sample size injector at 275 °C, the ratio of 10 % split to the column, 10 °C for column oven temperature for 3 min, 10 °C/min to 250 °C, the carrier gas of helium, the head pressure in the range of 70–86 kPa: (10–13 psig), and a sulfur chemiluminescence detector.

Triple repeat times were utilized to have reliable findings for BT content. S-content errors in the analysis with the maximum value below 1 % were applied.

The adsorption capacity was evaluated based on Eq. (1):

(1) $$AC=\frac{\left(C_{0}-C_{t}\right)*V_{S}}{m_{ads}}$$

where AC (mg g^−1^) is the adsorption capacity, C_0_ is the initial concentration of BT (ppm), C_t_ is the concentration of BT at time t, V is the volume of feed (L), and m is the mass of adsorbent per g.

### 2.6 Adsorbent revival process

At 150 °C, the adsorbent was thermally regenerated under N_2_ flow. An inner diameter of 1 cm and a length of 0.5 m stainless steel tube reactor was first used to place nanoadsorbent. The adsorbents were exposed to N2 flow at a specific rate for 2 h. The flow rate was regulated for the reactor by MFC (the mass flow controller, Bronkhorst). Lastly, desulfurization tests were performed using nanoasdorbents that had been regenerated.

### 2.7 Characterization

BrukerAXS-D8 Advance X-ray diffractometer was utilized for obtaining XRD patterns. Bruker Vertex 70 spectrometer recorded the FT-IR spectra with KBr pellets. PerkinElmer PYRIS Diamond was applied to perform the thermogravimetric analysis under an N_2_ atmosphere between 30 and 800 °C (the heat proportion of 10 °C/min). The adsorbents' morphology, size, and particle were recorded by FE-SEM microscopy (MIRA3 TESCAN-XMU microscope). Pore size and adsorption capacity were measured by physical sorption measurement. The Belsorp mini automatic adsorption instrument at 196 °C was used to obtain isotherms of N2 adsorption-desorption after the samples' degassing process 150 °C for 5 h.

## 3 Results and discussion

### 3.1 Characterization

**Fig. 1:**
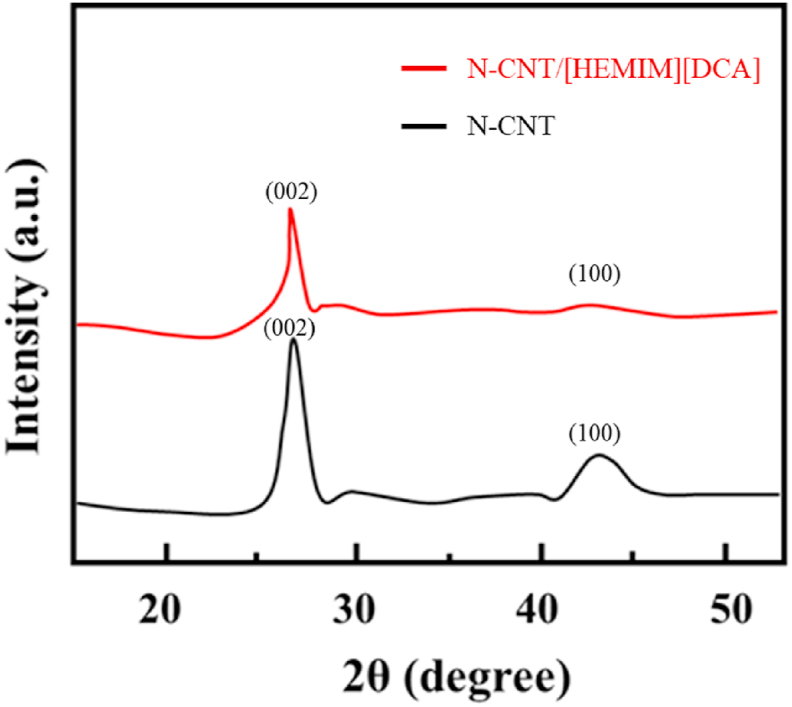
XRD of N-CNT and N-CNT/[HEMIM][DCA].

XRD patterns were utilized to analyze the crystalline properties of N-CNT and N-CNT/[HEMIM][DCA] (Fig. 1). Two prominent peaks can be seen at 26.08 (002 plane) and 44.13 (100 plane), which correspond to respective inter-planar spacing of 3.38 A° and 2.01 A°. N-CNT modification with IL slightly changes its crystallographic structure. On N-CNT/[HEMIM][DCA], the peaks moved to some extent to 26.01 and 44.02 (i.e., from 002 plane to 100 plane) with 3.39 A° and 2.08 A° inter-planar spacing, correspondingly. It is believed that the observed variation between pristine and modified N-CNT results from the crystallinity decrease of N-CNT treatment after modification [62,63].

**Fig. 2:**
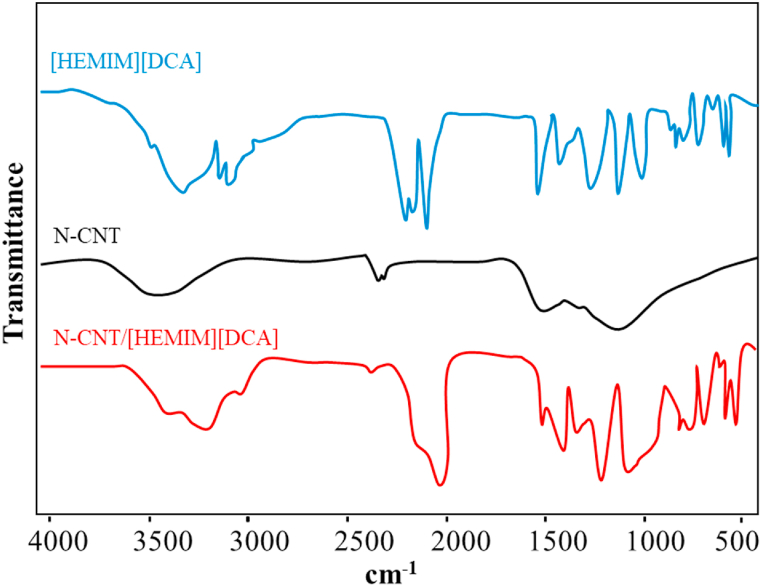
FTIR of N-CNT, [HEMIM][DCA], and N-CNT/[HEMIM][DCA].

In Fig. 2, FTIR spectroscopy defined N-CNT functionalities, [HEMIM][DCA], and IL loading on their surface. As shown in Fig. 2, the N-CNT pretreated showed the absorption peaks of 796 cm^−1^, 1120 cm^−1^, 1629 cm^−1^, and 1384 cm^−1^ assigned to pyrrolic N–H, graphitic C–N, pyridinic nitrogen and pyridinic oxide [32]. Besides, the characteristic peaks of amines, C]N, and OH groups for [HEMIM][DCA] were detected at 3318, 2200, 1569, and 1076 cm^−1^, respectively. FTIR analyses of pure N-CNT and [HEMIM][DCA] were compared to the pre-synthesized N-CNT/[HEMIM][DCA]. N-CNT/[HEMIM][DCA] exhibits no apparent shifts or variations, suggesting that neither component was affected by chemical interactions [64,65].

**Fig. 3:**
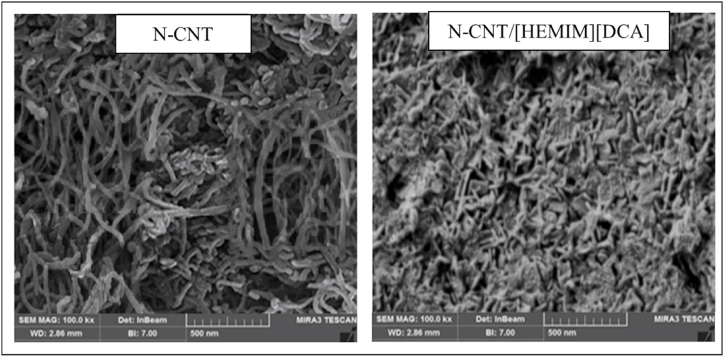
FE-SEM of N-CNT and N-CNT/[HEMIM][DCA].

Fig. 3 illustrates the morphologies of N-CNT, [HEMIM][DCA], and N-CNT/[HEMIM][DCA] hybrids. N-CNTs have a 15–25 nm outer diameter and a tubular morphology with a relatively plane surface. Due to the superior size of particles from 10 nm to 35 nm and the presence of N-CNT, the cubic and rod-shaped morphologies can be observed for N-CNT/[HEMIM][DCA] with separate harsh surfaces and curved edges. It has been observed that IL was well incorporated into N-CNTs in hybrids. The new morphology of the hybrid nanoadsorbents confirms the well-established interactions between investigated IL and N-CNTs, and N-CNTs' surfaces may also behave as the sites of formation and growth for hybrid nanoadsorbent. The FE-SEM images present N-CNT and N-CNT/[HEMIM][DCA] samples with monodispersity. In addition to displaying a relatively spherical shape and smooth surface, the N-CNT/[HEMIM][DCA] particles exhibit the same crystal structure as N-CNT, which could indicate that the N-CNT structure was retained while loading the IL [66]. Based on the surface morphology of both N-CNTs and N-CNTs–IL, it was found that adding IL did not drastically alter the morphology of CNT surfaces.

**Fig. 4:**
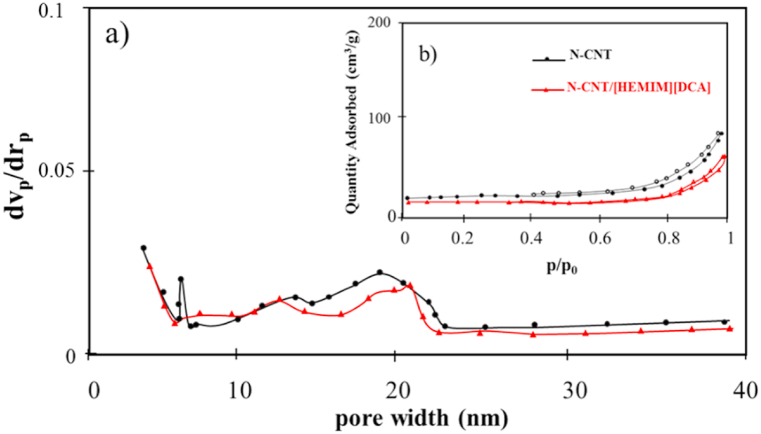
BET analysis of N-CNT and N-CNT/[HEMIM][DCA] a) pore-size distribution curves and b) nitrogen adsorption-desorption isotherm.

Adsorption processes depend heavily on the porosity of sorbents. Based on N_2_ adsorption-desorption isotherms, Table 1 and Fig. 4 show nanoadsorbent surface area and pore characteristics. N-CNT's specific surface area was 171.10 m^2^g^-1^, according to BET analysis. A slight surface area reduction was observed after modification and BT adsorption. IL could also block N-CNT pores as well as BT, resulting in a decrease in surface area. A slight decrease in average pore diameter was observed, with N-CNTs having 1.31 cm^3^ g^−1^. ILs may attach to N-CNT ends and defect sites, causing the decrease. IL modification of N-CNTs is thus confirmed.

**Table 1:** The results of BET analyses for surface area and pore volume of adsorbents.

Entry	BET Surface area (m^2^g^−1^) (before-after BT treatment)	Total pore volume (cm^3^ g^−1^) (before-after BT treatment)
**N-CNT**	171.10–160.25	1.31–1.21
**N-CNT/[HEMIM][DCA]**	167.85–133.42	1.19–1.02

**Fig. 5:**
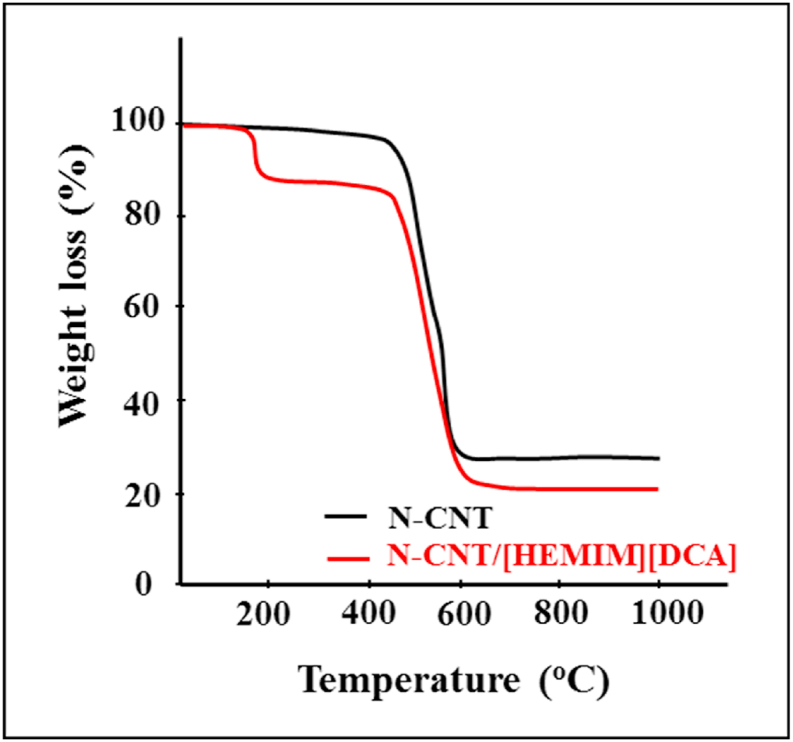
TGA of N-CNT and N-CNT/[HEMIM][DCA].

In high-temperature applications, porous materials are well-known for their thermal stability. Fig. 5 shows the TGA results of N-CNT and N-CNT/[HEMIM][DCA] composites. N-CNT@IL samples degrade at about 200 °C, whereas raw N-CNTs degrade at about 480 °C. As the functionalized IL on CNTs surface degrades at lower temperatures than CNTs, the mass loss for N-CNT-IL starts at 200 °C. This degradation accounts for the ∼13 % mass loss with the N-CNT-IL samples between 200 and 230 °C and the ∼5 % mass loss between 230 and 490 °C. N-CNT-IL samples exhibit a significant mass loss between 490 and 610 °C due to the degradation of the remaining N-CNT. In line with expectations, N-CNT samples degrade in a simple characteristic manner at 420 °C with a significant mass loss at 600 °C.

### 3.2 Effect of BT concentration, pH and temperature

**Fig. 6:**
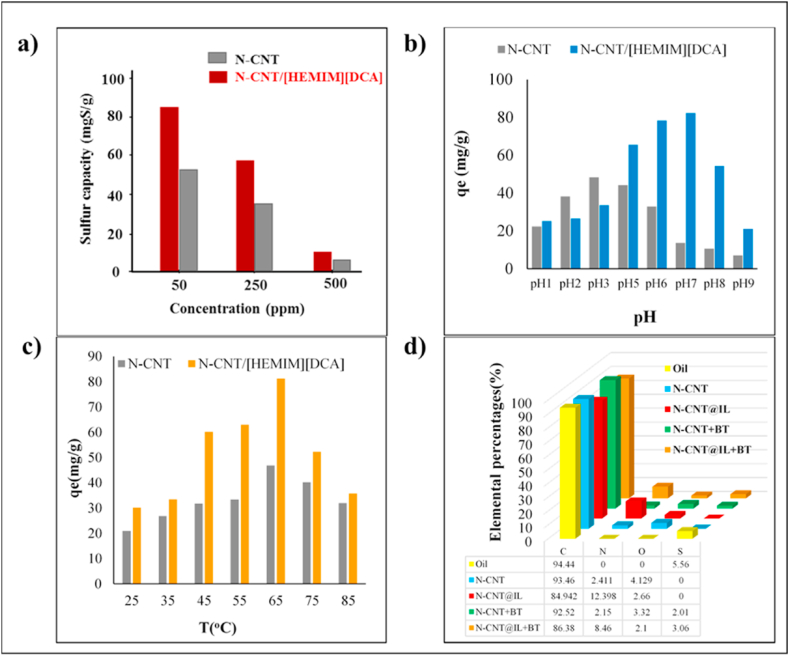
The effect of a) initial concentrations, b) pH, c) temperatures and d) elemental analysis on BT adsorption.

In Fig. 6a, the primary concentration of BT, ranging from 50 to 500 ppm, was investigated in the desulfurization process. The adsorption capacity of N-CNT and N-CNT/[HEMIM][DCA] was 54.3 mg/g and for 83.6 mg/g for 50 ppm BT, respectively. As the initial BT concentration increased, the removal percentage reduced. When BT concentrations are high, nanoadsorbents are saturated by BT. A nanoadsorbent requires a great surface area, an available interior capacity, and a promising opening dimension to be effective. Furthermore, the pores in the adsorption process can enormously enhance the adsorption of the adsorbate. Nanoadsorbents prepared from micropores and mesopores were found to have effluent active sites on their surfaces, according to BET analysis. Due to this, an increase in BT concentration resulted in BT occupying sorbent active sites and pores, decreasing the performance of nanoadsorbents. The effect of pH and temperatures was also investigated (Fig. 6 b and c). It is shown that the optimum pH for N-CNT and N-CNT/[HEMIM][DCA] is pH 3 (48.32 mg/g) and pH 7 (82.23 mg/g), respectively. Also, the highest adsorption capacity can be observed at 65 °C for both nanoadsorbents (Fig. 6 c). Fig. 6 d shows the results of elemental analysis of nanoadsorbents before and after desulfurization. It is obvious that the percentages of sulfur content in N-CNT/[HEMIM][DCA] is higher than N-CNT due to its higher adsoption capacity.

### 3.3 Adsorption isotherms

The interaction between BT and the surfaces of the adsorbent was described with adsorption isotherms. As a result, the adsorption isotherms were utilized to evaluate the adsorption capacity. The Langmuir isotherm (Eq. (2)) and Freundlich isotherm (Eq. (3)) are utilized to determine the maximum adsorption capacity for BT and the constants of equilibrium isotherms. These constants can determine adsorbent properties, mechanisms, and affinity. Langmuir and Freundlich are the most common models. These simple models can explain experiments in different concentration ranges [3,67].

(2) $$\frac{C_{e}}{q_{e}}=\frac{C_{e}}{q_{m}}+\frac{1}{bq_{m}}$$

(3) $${q_{e}=K}_{f}{C_{e}}^{1/n}$$

According to the Langmuir model, the behavior of an adsorbate as an ideal gas is explained by monolayer adsorption at isothermal conditions. The equilibrium adsorption capacity, equilibrium concentration BT, extreme adsorption capacity on a single layer, and Langmuir constant are represented by q_e_, C_e_, q_m_, and b. Additionally, the equilibrium parameter (RL) indicates the nature of the adsorption process (Eq. (4)).

(4) $$R_{L}=\frac{1}{1+K_{L}C_{i}}$$

RL of 0–1 shows favorable adsorption and unfavorable for higher values. The irreversible or linear adsorptions are assigned to RL = zero and one, respectively. In the Freundlich model (Eq. (3)), the heterogeneity of surface and distribution of active sites are present exponentially and non-uniformly. A higher q_e_, c_e_, and k_f_ value indicates a greater equilibrium adsorption capacity (mg/g), equilibrium BT concentration (mg/l), and Freundlich parameter.

**Fig. 7:**
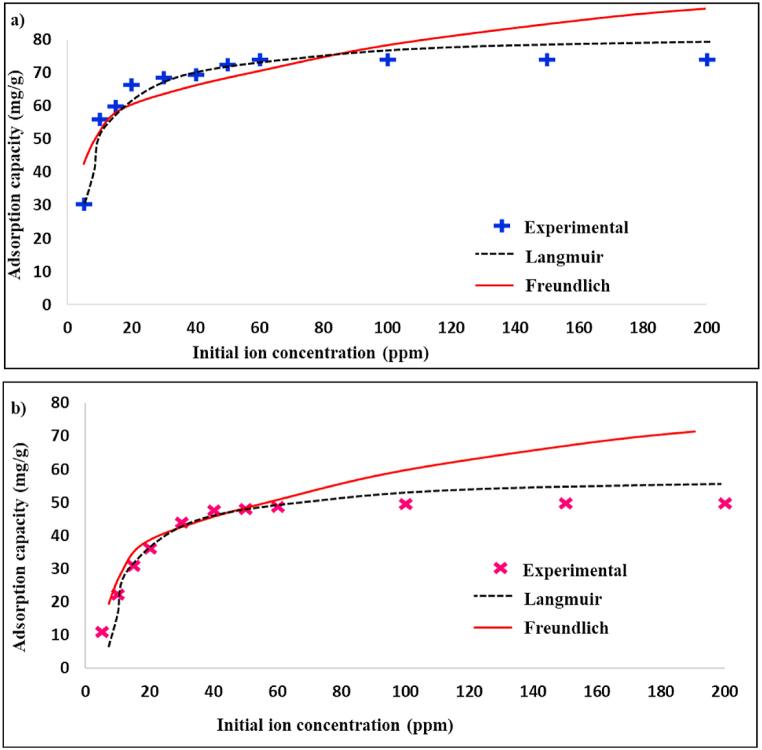
BT adsorption based on a) Langmuir and b) Freundlich models.

Table 2 provides the calculated adsorption parameters of the nanoadsorbents. In Fig. 7 (a,b), q_e_ vs. C_e_ shows that the adsorption process for N-CNT/[HEMIM][DCA] and N-CNT follows the Langmuir model. Furthermore, all R_L_ values were between 1-0, indicating a favorable adsorption process.

**Table 2:** Adsorption parameters of Langmuir and Freundlich models.

adsorbent	Langmuir			Freundlich		
	q_max_	b	R^2^	K_F_	n	R^2^
N-CNT	54.3	0.010	0.92	2.95	1.68	0.89
N-CNT/[HEMIM][DCA]	83.6	0.015	0.98	4.76	1.96	0.94

### 3.4 Effect of adsorption time

**Fig. 8:**
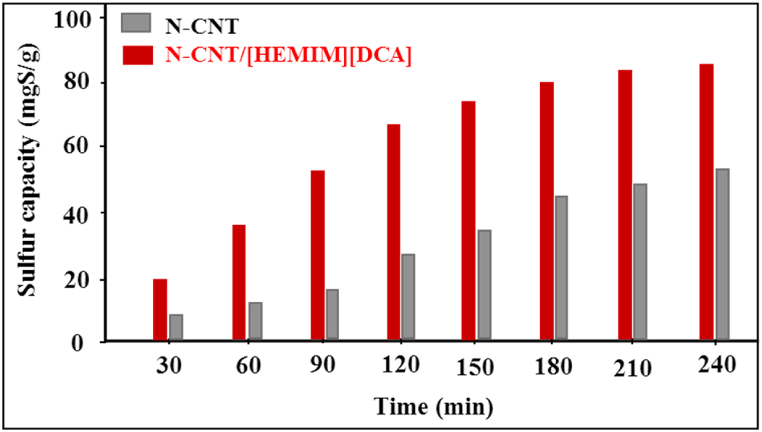
BT adsorption based on different contact time.

To understand BT adsorption's mechanism, pseudo-first and second-order kinetic models were applied [68]. Nanoadsorbents were supplemented to a model fuel solution containing an initial 50 ppm concentration for BT. A regular time interval was used to measure output BT concentrations (Fig. 8).

Nanoadsorbents' kinetic characteristics were investigated by pseudo first- and second-order equations, Eq. (5) and Eq. (6), respectively. Porosity type, particle size, kinesis in the solution phase, and hydrodynamics of contacts between particles can affect the adsorption rate for an explicit molecule. According to Table 3, the adsorbents' kinetic parameters are calculated as q_t_ (mg/g), q_e_ (mg/g), and k_1_ and k_2_ (g/mg.min) at t (min) [69,70].

(5) $${q_{t}=q}_{e}\left(1-e^{-k_{1}t}\right)$$

(6) $$q_{t}=\frac{k_{2}{q_{e}}^{2}}{1+k_{2}q_{e}t}t$$

**Table 3:** Adsorption factors of pseudo-first- and second-order models

Adsorbent	q_eexp_	Pseudo first order			Pseudo second order		
		q_max_	b	R^2^	K_F_	n	R^2^
N-CNT	58	84.8	0.064	0.89	83.5	2.2*10^−4^	0.90
N-CNT/[HEMIM][DCA]	79	66.6	0.021	0.94	97.1	0.0210	0.99

**Fig. 9:**
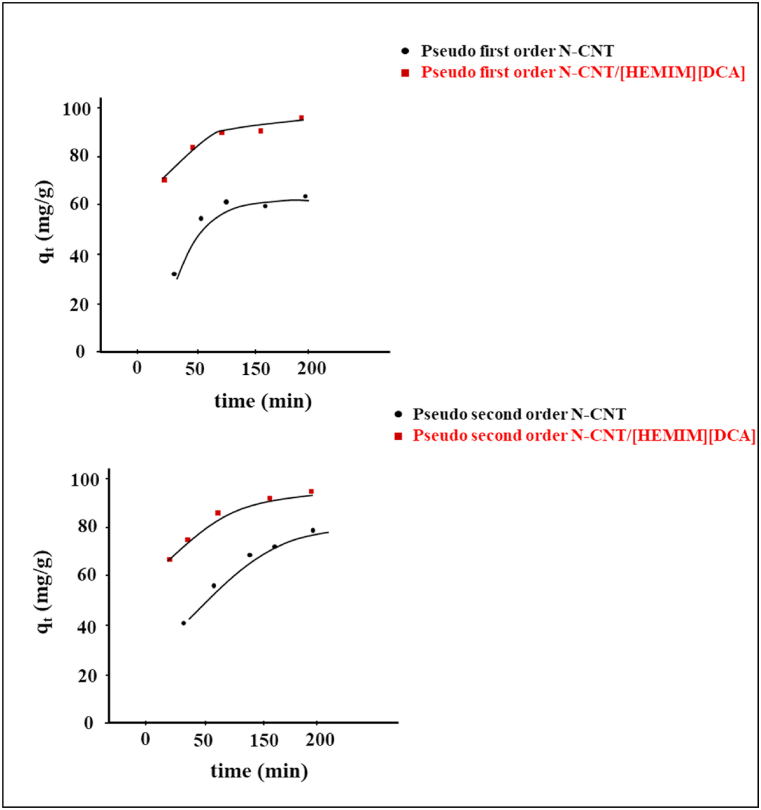
The pseudo-first- and second-order models for BT adsorption.

Based on experimental results, the adsorption ratio followed a second-order model with R^2^ of 0.99, a better-resigned value (Fig. 9). The pseudo–second-order kinetic model, in accordance with its underlying assumption, posits that the rate-limiting step is attributed to chemical sorption or chemisorption. This model provides predictions that encompass the entire spectrum of adsorption behavior. In this particular scenario, it is crucial to note that the rate of adsorption is contingent upon the adsorption capacity rather than the concentration of the adsorbate.

Table 3 displays the theoretical measurement agreement of q_t_ with the experimental values. Thus, the pseudo-second-order model fits the BT sorption on N-CNT/[HEMIM][DCA].

### 3.5 Results of thermodynamic studies and temperature's effect

Temperature affects adsorption equilibrium, thermodynamic factors, and adsorption energy. BT diffusion into the adsorbent's surface and pores can be sped up by temperature increase. Therefore, adsorption capacity at 35, 45, and 60 °C was investigated. The thermodynamic characteristics such as enthalpy change (ΔH, kJ/mol), adsorption equilibrium constant (K_0_), entropy change (ΔS, J/mol.K), and free energy change (ΔG, kJ/mol) for the adsorption of BT were considered as Eq. (7)−10 [[71], [72], [73]].

(7) $$K_{p}=\frac{y_{s}C_{s}}{y_{e}C_{e}}$$

(8) $$\Delta G=-RT\;\ln\;\left(K_{p}\right)$$

(9) $$\ln\left(K_{p}\right)=\frac{-\Delta H}{RT}+\frac{\Delta S}{R}$$

(10) $$K_{p}=\frac{C_{s}}{C_{e}}$$

where, Cs (mg/g) is the adsorbed BT concentration, R is the gas constant (R = 8.314 J/mol.K), Ce (mg/L) is BT concentration at equilibrium, and T is the temperature (K). The Kp values were gotten by ln (Cs/Ce) vs Cs curve and generalizing Cs to zero.

**Fig. 10:**
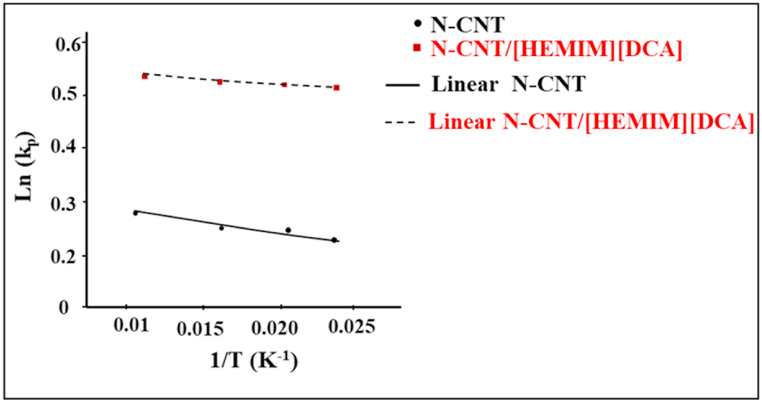
lnKp against 1/T curves

The ΔH and ΔS values were obtained using lnKp against 1/T curve (Fig. 10). Table 4 shows the ΔG, ΔH, and ΔS values. The ΔG negative values indicate BT spontaneous adsorption. As the proportional relative of adsorption capacity and temperature, the BT and nanoadsorbents interactions were endothermic. The physical adsorption process is generally considered for H_ads_ lower than 40 kJ/mol. In other words, physical adsorption can be performed between BT and nanoadsorbents of N-CNT and N-CNT/[HEMIM][DCA]. The ΔS positive amount showed a higher degree of adsorption freedom.

**Table 4:** Calculated ΔG, ΔH, and ΔS values of adsorption of BT on nanoadsorbents.

Adsorbent	ΔH (KJ/mol)	ΔS (KJ/mol.K)	R^2^	ΔG (KJ/mol)		
				35 °C	45 °C	60 °C
N-CNT	29.36	2.07	0.95	−0.76	−0.86	−0.98
N-CNT/[HEMIM][DCA]	36.87	6.40	0.99	−1.43	−1.74	−1.88

### 3.6 Assessment of BT removal

The N-CNT/[HEMIM][DCA] adsorption capacity was superior to that of the pure N-CNT. The adsorption capacity was directly affected by the primary sites of N-CNT. Basic sites must be present to improve the adsorption act of N-CNT/[HEMIM][DCA]. A synergetic effect between N-CNT and [HEMIM][DCA]might also contribute to the upsurge in BT adsorption capacity on N-CNT/[HEMIM][DCA]. As a result of the improved adsorption heat for the BT adsorption of N-CNT/[HEMIM][DCA], the heat of adsorption for N-CNT/[HEMIM][DCA]was 36.87 kJ/mol, compared to 29.36 kJ/mol for pure N-CNT. Compared to powder adsorbents, nanoadsorbents with favorite morphologies have more direct contact. [HEMIM][DCA] growing on the N-CNT increased the interactions (BT and adsorbent) [66,74]. Table 5 indicates an assessment list of desulfurization capacities for numerous adsorbents.

**Table 5:** Comparing the efficiency of sulfur adsorption for several adsorbents.

sorbent	Pollutant	Initial concentration (ppm)	T (°C)	Capacity (mg S/g)	Ref.
CuCe mesoporous Y zeolites	benzothiophene and dibenzothiophene	1000	25	45	[75]
MOF-199	sulfur component	400	30	40	[76]
iron-alumina composites	dibenzothiophene	1500	25	57	[77]
Microwave synthesized carbon nanotube (M-CNT), and carbon nanofiber (M-CNF)	**dibenzothiophene**	250	30	35%–43 %	[33]
N-CNT/[HEMIM][DCA]	benzothiophene	500		83.6	This work

### 3.7 Adsorption mechanism

**Fig. 11:**
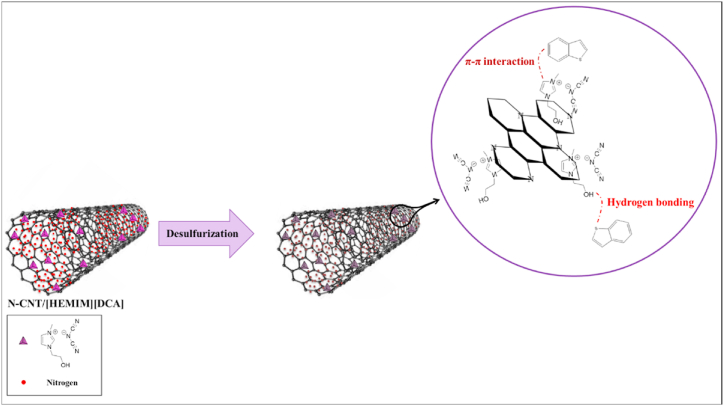
Mechanism of BT adsorption on N-CNT/[HEMIM][DCA].

**Fig. 12:**
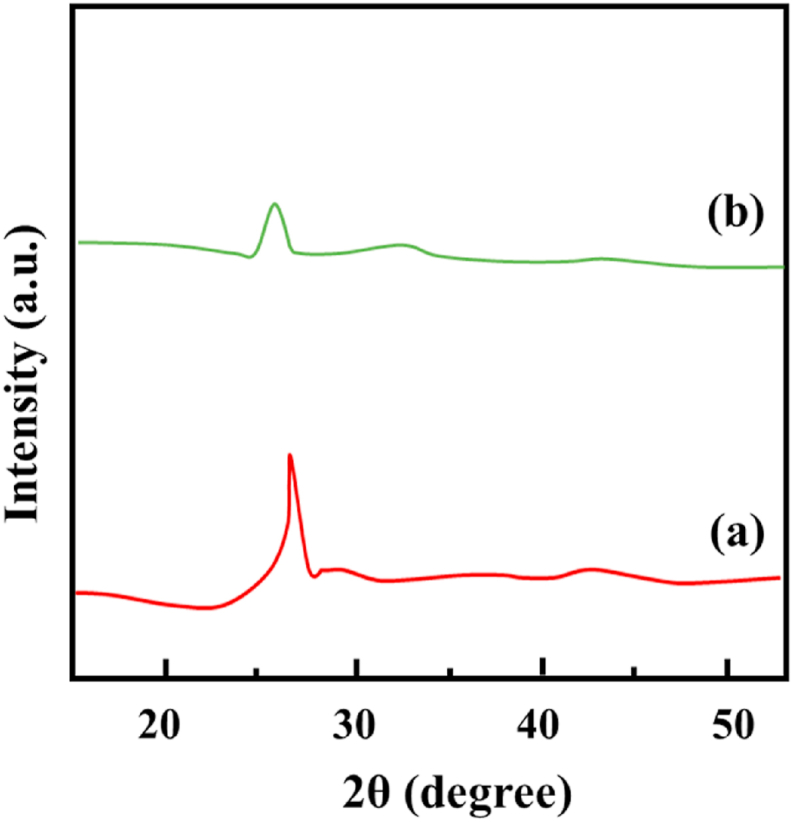
XRD patterns of N-CNT/[HEMIM][DCA] a) before and b) after the BT adsorption.

Due to the two-electron pairs of sulfur having a six-electron π system, BT can be a π-type donor to form a π-complex with adsorbent [78]. Imidazole moiety's great polarizable element density to have stronger π– π interaction [53,79,80]and the BT adsorption mechanism on IL can be credited to a possible combination of aromatic sulfides and imidazoles. According to Fig. 11, the BT adsorption mechanism on N-CNT/[HEMIM][DCA] can be seen. Adsorption occurs when particles diffuse into the interior adsorbent surface. As elements diffuse into the adsorbent's internal surface, diffusion rates control the adsorption ratio. Due to augmented confrontation and reduced motivating force, the adsorption ratio decreases with time. N-CNT/[HEMIM][DCA] was initially adsorbed with BT onto its micro- and mesoporous adsorptive positions. Over time, the adsorption reduced as the pores became saturated. The occurrence of N-doped locations such as pyridinic oxide and pyridinic) importantly enhanced BT's desulfurization process [81,82]. The nitrogen dopants could enhance the polarity of N-CNTs, improving their chemical reactivity. In general, polar adsorbents provide a better adsorption capacity and are better suited to adsorption. BT can be reacted with nanosized N-CNT/[HEMIM][DCA] at low temperatures, and their great surface area can deliver extra dynamic sites. Nanoadsorbents with both properties improve desulfurization performance significantly. In contrast, increasing the temperature (35–60 °C) enhances adsorption. Adsorption performance is effectively influenced by increased temperature. As a result, physisorption dominates adsorption processes and chemisorption occurs only very gently. XRD spectra of N-CNT/[HEMIM][DCA] in Fig. 12 (a,b) show no significant changes for the peak positions, but there is a slight decrease in intensities for the spent sample, which may indicate physisorption-mediated adsorption.

[HEMIM][DCA] addition to the N-CNT provided an abundance of active sites and brought about the N-CNT absorbency for BT. Pure N-CNT consists mainly of micropores, which are effective at attracting BT but are quickly saturated. By defecting the N-CNT crystals, [HEMIM][DCA] could form new mesopores and alter the pore size distribution. This makes the adsorption process more efficient, and the adsorption capacity is further improved. N-CNT adsorption energy was greatly enhanced due to the adsorption energy enhancement of carbon-nitrogen bonds and oxides of pyridinic and pyridinic moieties. In N-CNT/[HEMIM][DCA] nanocomposite, the BT adsorption capacity was improved by engineering the permeability and nature of the active sites.

### 3.8 Zeta potential

**Fig. 13:**
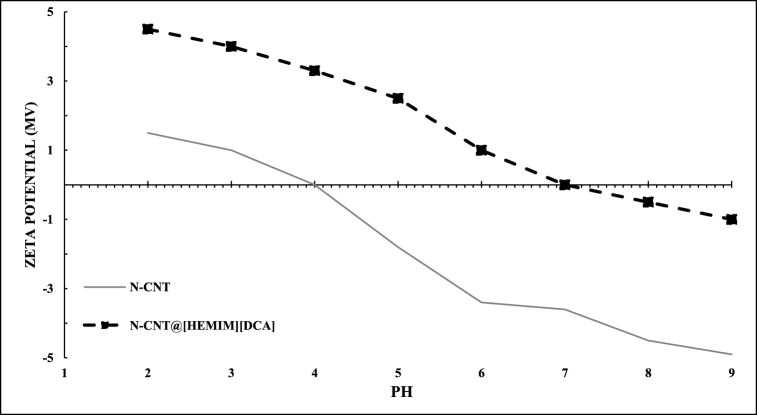
Zeta potentials for N-CNT and N-CNT/[HEMIM][DCA].

Zeta potential (ZP) is one of the most important parameters to determine the stability of a colloidal system. The dependence of ZP on pH for nanoabsorbents and BT is shown in Fig. 13. It can be seen that the N-CNT sample reached negative ZP values in almost all the studied pH ranges. The highest value of ZP (1.50 mV) was observed at pH 2. With increasing pH, ZP reached less positive values and the isoelectric point (IEP) was determined at pH 4. The surface charge of synthesized N-CNT/[HEMIM][DCA] was evaluated using zeta potential analysis. The presence of hydroxyl and organic groups in the ionic liquid section increases the sensitivity to pH in the adsorbent. As shown in Fig. 13, with increasing pH from 2 to 9, the value of zeta potential decreased from +4.50 mV to −1.00 mV. In comparison, in N-CNT modified by ionic liquid, all ZP values were shifted to more positive regions and IEP was detected at pH 7.

### 3.9 Nanoadsorbent lifetime

**Fig. 14:**
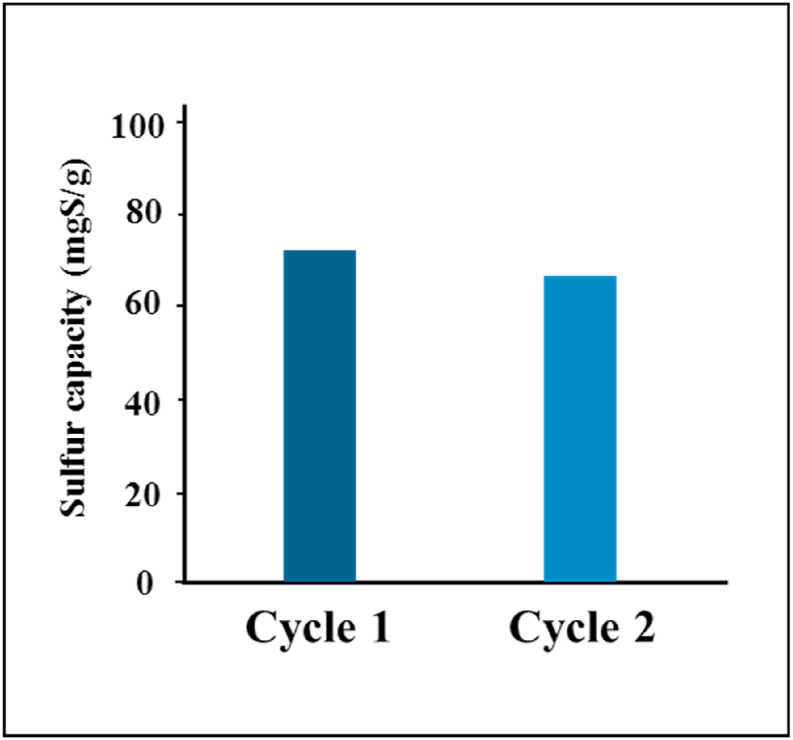
Cycles of regeneration for N-CNT/[HEMIM][DCA].

N-CNT/[HEMIM][DCA] nanoadsorbent regeneration is illustrated in Fig. 14. According to TGA analysis, the thermal revival was directed at 160 °C under N_2_ flow. After nitrogen sweeping at 160 °C for 2 h followed by heating at 120 °C for 4 h in a vacuum, N-CNT/[HEMIM][DCA] was saturated with BT and became darker. After two regeneration cycles with N-CNT/[HEMIM][DCA], the adsorption capacity decreased to about 25 %, and the sulfur removal capacity reached about 62.5 mg/g. N-CNT/[HEMIM][DCA]showed good recyclability and potential as a promising BT adsorbent.

## 4 Conclusion

To improve the performance of sulfur adsorbents, in this study, N-doped CNTs were modified by imidazolium-based IL 1-(2-hydroxyethyl)-6-methylimidazolium dicyanamide [HEMIM][DCA], resulting in a green hybrid, N-CNT/[HEMIM][DCA]. Through adsorptive desorption, the hybrid product was used to desulfurize BT. It was found that the modification of N-CNT with IL slightly alters its crystallography in the third step. N-CNT/[HEMIM][DCA] does not exhibit any apparent shifts or variations in FTIR analysis, suggesting that neither component was affected by chemical interactions. Observations of the surface morphology of N-CNTs and N-CNTs–IL indicated that IL did not significantly alter CNT surfaces' morphology. A slight reduction in average pore diameter was observed, with N-CNTs having 1.31 cm^3^ g^−1^. The decrease may be caused by ILs attaching to N-CNT ends and defect sites. It confirms the IL modification of N-CNTs. Accordingly, N-CNT samples degrade at 420 °C with a significant mass loss at 600 °C as the initial BT concentration increases. Nanoadsorbents are saturated with BT at high BT concentrations.

Adsorption isotherms provided an understanding of how BT interacts with sorbent surfaces. As a result, the isotherms of adsorption models were used to study adsorption capacity. Langmuir's model has a higher R^2^ value than Freundlich's, indicating monolayer coverage of the BT onto the N-CNT. Besides, the pseudo-second-order model fits the BT sorption on N-CNT/[HEMIM][DCA]. Also, adsorption capacity at 35, 45, and 60 °C was investigated. As the adsorption capacity and temperature are correlated proportionally, the interaction between BT and nano adsorbents was endothermic. Physical adsorption can occur between BT and nanoadsorbents such as N-CNT and N-CNT/[HEMIM][DCA]. N-CNTs' primary sites directly influenced their adsorption capacity. Compared with pure N-CNT, the heat of adsorption for N-CNT/[HEMIM][DCA] was 36.87 kJ/mol, due to the enhanced heat of adsorption for BT adsorption. The adsorption capacity of N-CNT/[HEMIM][DCA] decreased to about 25 % after two regeneration cycles, and sulfur removal capacity reached about 62.5 mg/g after two regeneration cycles. There is potential for N-CNT/[HEMIM][DCA] to be a promising BT adsorbent due to its high recyclability.

## Data availability statement

The authors are unable or have chosen not to specify which data has been used.

## CRediT authorship contribution statement

**Seyed Mohammad Reza Shoja:** Writing – original draft, Methodology, Data curation. **Majid Abdouss:** Validation, Supervision, Project administration. **Raheleh Saeedirad:** Writing – review & editing, Methodology, Formal analysis.

## Declaration of competing interest

The authors declare that they have no known competing financial interests or personal relationships that could have appeared to influence the work reported in this paper.
